# Time-domain based evaluation of detection efficiency in liquid scintillation counting

**DOI:** 10.1038/s41598-021-91873-1

**Published:** 2021-06-14

**Authors:** Krasimir Mitev, Chavdar Dutsov, Philippe Cassette, Benoît Sabot

**Affiliations:** 1 Faculty of Physics, Sofia University “St. Kliment Ohridski”, 1164 Sofia, Bulgaria; 2 Université Paris-Saclay, CEA, LIST, Laboratoire National Henri Becquerel (LNE-LNHB), 91120 Palaiseau, France

**Keywords:** Techniques and instrumentation, Experimental nuclear physics

## Abstract

This work explores the distribution of time intervals between signals from the photomultiplier tubes (PMTs) of a liquid scintillation counting (LSC) system when a scintillation burst caused by an ionizing particle is detected. This distribution is termed the cross-correlation distribution and it is shown that it contains information about the probability to detect a scintillation event. A theoretical model that describes the cross-correlation distribution is derived. The model can be used to estimate the mean number of detected photons in a LSC measurement, which allows the calculation of the detection efficiency. The theoretical findings are validated by Monte Carlo simulations and by experiments with low-energy beta-emitting and electron-capture radionuclides ($^3\hbox {H}$, $^{14}\hbox {C}$, $^{63}\hbox {Ni}$ and $^{55}\hbox {Fe}$), with dedicated LSC systems and several commercial LSC cocktails. The results show that some of the parameters of the cross-correlation distribution such as the peak height or the kurtosis can be used as detection efficiency estimators or quenching indicators in LSC. Thus, although the time domain and the cross-correlation distribution have received little to no attention in the practice of LSC, they have the capacity to bring significant improvements in almost all LSC applications related to activity determination of low-energy beta-emitting and electron-capture radionuclides. The results also suggest concepts for the development of innovative LSC systems.

## Introduction

Liquid scintillation counting (LSC) is a powerful technique for the measurement of ionizing radiations. It finds applications in many areas such as: radioactivity survey in the environment or in nuclear facilities^1^, radionuclides standardization^2^, high energy and neutrino physics^3^, $$^{14}$$C dating^4^, marine studies^5^, environmental studies and monitoring^6^. Generally, LSC analyzers are instruments which employ two or three photomultiplier tubes (PMTs) connected to coincidence counting circuits. The physical quantities used to extract information about the measured radioisotope are the experimental coincidence counting rates and pulse-height spectra. These quantities are used in methods for primary activity standardization, like the Triple to Double Coincidence Ratio (TDCR) method or the CIEMAT/NIST efficiency tracing (CNET) method as well as in many protocols for the determination of activity of various isotopes^7^.

The TDCR method has been developed for the direct activity measurement of beta and electron capture radionuclides, including the radionuclides decaying towards the ground level of the daughter^2^. It uses a statistical model which is applied to the counting rate data from a LSC system with three PMTs. The CNET method was also developed for radionuclide standardization using a tritium standard source. Both methods are based on the free parameter model, in which the detection efficiency is derived from the mean number of detected photons per decay. The time domain information and the distribution of the time differences between the responses of the PMTs has received almost no attention and has not been used so far.

Activity measurements by LSC methods require the radioactive sample to be dissolved in a scintillation cocktail. This generally ensures high detection efficiency as the ionizing particles are emitted directly in the cocktail. However, the addition of solutes to the cocktail may be a source of sample variability and may reduce the efficiency by means of chemical or color quenching. Thus, as the radioactive source is a part of the detection system, the sample preparation procedures and the evaluation of counting efficiency are of paramount importance in LSC. In routine analysis using commercial LSC counters, there are various approaches for detection efficiency estimation and quench correction. Typically, the pulse height spectra of the sample with and without an external gamma-ray source are acquired and the efficiency is estimated through quench indicating parameters—e. g., the H number (H#), the Spectral quench parameter of the external standard SQP(E)^7^, the Spectral index of the transformed external standard spectrum tSIE^7^, etc. The application of these methods requires careful preparation of a set of calibration sources with known activity and different levels of quenching. It also requires the establishment of efficiency calibration curves. Most of the methods used for efficiency calibration in LSC are based on pulse-height spectra analysis^8^ and the time domain information has not been used in the methods developed so far.

The objective of this work is to introduce the cross-correlation distribution for LSC systems with two or three PMTs and to demonstrate that it contains information about the detection efficiency. A theoretical model is developed in which the cross-correlation distribution is derived. It is shown how the model can be used to estimate the mean number of detected photons in a given measurement. The theoretical findings are validated by Monte Carlo simulations and supported by experimental data from dedicated experiments with low-energy beta-emitting and electron-capture radionuclides and commonly used commercial LSC cocktails. The results of this work imply that the measured cross-correlation distribution can act as an efficiency estimator or a quenching indicator in LSC. These results have long-reaching consequences as they allow the determination of the counting rate and the detection efficiency from data acquired in a LSC measurement.

## Results

### Theoretical

**Figure 1:**
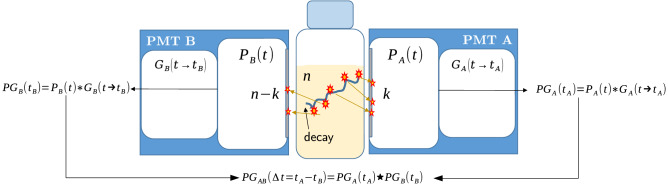
Schematic description of a two PMT LSC system, the considered processes in the time domain and the basic equations that describe them. The symbols $\star$ and $*$ indicate the cross-correlation and convolution operators, respectively.

In order to explore the time domain information in LSC, we will derive the distribution of time intervals between the signals from the PMTs when they detect scintillation burst caused by an ionizing particle. This distribution arises from the cross-correlation of the time responses of the PMTs (see Fig. 1) and, hiring the term from the signal processing field, will be referred to as the cross-correlation distribution. The basic assumptions and the main steps behind its derivation are outlined below.

The following sequence of processes in a two PMT system (Fig. 1) will be considered without loss of generality. A decay of a radioactive isotope within the scintillation cocktail leads to the deposition of energy *E* in the liquid scintillator, creating excites states of the solvent. The energy transfer of these excited states to the fluorescent molecules could produce light by radiative de-excitation. It is considered that *n* of the emitted photons are detected by the PMTs with *k* photons being detected in one PMT and $n-k$ in the other.

For the purpose of this work a primary event in a PMT is defined as the detection of the first photon, out of total *k* photons detected by that PMT, for a given scintillation burst. This definition is an adequate model as the primary event forms the rising edge of the PMT signal and its timestamp is recorded by the analyzing electronics. The following assumptions will be made hereafter:

- It is considered that the time dependence of the light emission of the liquid scintillator is described by a single exponential distribution of the type: $p(t)=\lambda e^{-\lambda t}$, where $\lambda$ is the decay constant of the scintillator. Thus, the finite pulse rise time and the delayed scintillation component of the scintillator will not be considered hereafter. This makes sense as the creation of initial excited species in the scintillator is a very fast process.
- The transport of photons from the point of emission to the photo-cathode is considered immediate. Thus, possible changes in the time sequence due to photon travelling in the optical chamber of the detector are neglected. In a typical LSC system, the source diameter is a few centimeters and the photon delays in the system are in the order of a hundred to a few hundred picoseconds.
- It is also considered that the first photon that creates a photo-electron at the photo-cathode of the PMT will be the first one detected by the electronics. Thus, a possible rearrangement of the photon detection sequence due to the multiplication process in the PMT is considered with low probability and of minor importance and is neglected.
- The time response of the PMTs $G_A(t \rightarrow {} t_A)$ is considered to be a Gaussian distribution.

The quantity of interest in this work is the probability distribution $D(\Delta t)$ of the time intervals between the primary events in a two PMT detection system, which is given by:

1 $$\begin{aligned} D(\Delta t; \varphi , \lambda , \epsilon _A, \epsilon _B, \mu , \sigma ) = \frac{1}{L} {\underbrace{ \int _0^{\text {E}_\text {{max}}} S(E)}_{\text {Energy spectrum}}} ~{\underbrace{ \sum _2^\infty \frac{\left( {\bar{n}}(E; \varphi )\right) ^n}{n!} e^{-{\bar{n}}(E; \varphi ) }}_{\text {Poisson statistics}}} ~{\underbrace{ \sum _{k = 1}^{n-1} \left( {\begin{array}{c}n\\ k\end{array}}\right) \epsilon ^k_A\epsilon ^{n-k}_B}_{\text {Combinatorial term}}} ~{\underbrace{ A (\Delta t; n, k, \lambda , \mu , \sigma )}_{\text {Cross-correlation}}}~dE, \end{aligned}$$

where $E_\text {max}$ is the maximum energy in the particle spectrum *S*(*E*), $\mu$ is the difference in the static time delay of the two PMTs and $\sigma$ is the quadratic sum of the standard deviation of the Gaussian time jitters in the two PMTs. *L* is a normalization coefficient equal to the probability of all detected events:

2 $$\begin{aligned} L = \int _0^{\text {E}_\text {{max}}} S(E) \sum _2^\infty \frac{\left( {\bar{n}}(E; \varphi )\right) ^n}{n!} e^{-{\bar{n}}(E; \varphi )} \sum _{k = 1}^{n-1} \left( {\begin{array}{c}n\\ k\end{array}}\right) \epsilon ^k_A\epsilon ^{n-k}_B~dE, \end{aligned}$$

which is necessary because events with less than two detected photons will not lead to coincidence and will not be detected. The Poisson distribution accounts for the probability to have *n* detected photons in a given decay if there are ${\bar{n}}$ photons detected on average for a particular energy. The mean number of detected photons with respect to the deposited energy in the cocktail *E* can be obtained as:

3 $$\begin{aligned} {\bar{n}}(E; \varphi ) = E Q(E) \varphi , \end{aligned}$$

where *Q*(*E*) is a factor that takes into account the ionization quenching and is dependent on the energy of the particle as well as on the stopping power of the particle in the scintillator. The parameter $\varphi$ is called *figure of merit* (*fom*) and it is equal to the mean number of detected photons per keV of effective energy released into the scintillator—i.e., after taking into account ionization quenching. The most widely used description of the ionization quenching function *Q*(*E*) is given by Birks’ semi-empirical equation^9^:

4 $$\begin{aligned} Q(E) = \frac{1}{E} \int _0^E \frac{dE}{1 + kB(dE/dx)}, \end{aligned}$$

where *dE*/*dx* is the stopping power of the particle and *kB* is the Birks parameter, specific to the used scintillation cocktail.

The cross-correlation term $A(\Delta t)$ gives the probability to have a given $\Delta t$ between the first photon from *k* total detected in one PMT and the first photon from $(n-k)$ total detected in the other PMT. It is given by:

5 $$\begin{aligned} A(\Delta t; n, k, \lambda , \mu , \sigma ) = \frac{1}{n} \Bigl ( k~EMG_{AB}\left( \Delta t; (n-k)\lambda , \mu , \sigma \right) + (n-k)~EMG_{BA}\left( -\Delta t; k\lambda , -\mu , \sigma \right) \Bigr ), \end{aligned}$$

where *EMG* stands for the exponentially modified Gaussian distribution which is a convolution between an exponential and a Gaussian distribution and has the form:

6 $$\begin{aligned} EMG(\Delta t; \tau , \mu , \sigma ) = \frac{\tau }{2} e^{\frac{\tau }{2} \left( 2\mu + \tau \sigma ^2-2\Delta t\right) } \text {erfc}\left( \frac{\mu + \tau \sigma ^2 - \Delta t}{\sqrt{2}\sigma } \right) . \end{aligned}$$

The parameters $\mu$ and $\sigma$ are the Gaussian centroid and standard deviation, respectively and $\tau$ is the decay constant of the exponential distribution. The two terms in the sum in Eq. (5), $EMG_{AB}$ and $EMG_{BA}$, consider the two cases $t_A - t_B \ge 0$ and $t_A - t_B \le 0$. The full derivation of Eqs. (1) and (5) is given in “Methods”. There, a similar equation is also derived for a three-PMT detection system.

Equation (1) is remarkable because it shows that the cross-correlation is a function of the *fom* $\varphi$. This implies that, in principle, if the decay constant of the scintillator $\lambda$ and the time response properties of the system ($\mu , \sigma$, $\varepsilon$) are known the value of $\varphi$ can be estimated from the cross-correlation distribution. The *fom* is a key parameter because it gives the possibility to calculate the detection efficiency and, from it, the activity of the sample. The detection efficiency for coincidences in a two PMT system $\varepsilon _{\text {AB}}$ is given by the free parameter model in LSC^8^:

7 $$\begin{aligned} \varepsilon _\text {AB} = \displaystyle \int _0^{E_\text {max}} S(E) \left( 1-e^{\frac{-{\bar{n}}(E;\varphi )}{2}}\right) ^2dE, \end{aligned}$$

where the factor 2 stays for the number of PMTs in the system. The only free parameter that needs to be determined in order to calculate the detection efficiency is $\varphi$. A similar equation is derived for a three PMT system. By knowing the detection efficiency, the activity of the sample *A* is calculated as:

8 $$\begin{aligned} A = \frac{n_\text {AB}}{\varepsilon _\text {AB}}, \end{aligned}$$

where $n_\text {AB}$ is the net (background corrected) counting rate of the two PMTs in coincidence.

The value of $\varphi$ can also be used to determine the average number of detected photons ${\bar{n}}$ in the case of measurements of nuclides with an energy spectrum *S*(*E*):

9 $$\begin{aligned} {\bar{n}} = \int _0^{E_\text {max}} S(E)~EQ(E)\varphi ~dE. \end{aligned}$$

The correct calculation of the energy spectrum *S*(*E*) is very important for the radionuclide standardization by LSC and for many beta-emitters the reliability of the beta-spectra calculation was carefully evaluated^10,11^. For a given energy spectrum and ionization quenching function, the relationship between the *fom* and the average number of detected photons ${\bar{n}}$ is unambiguous so, the knowledge of either $\varphi$ or ${\bar{n}}$ is sufficient to determine the detection efficiency and thus the activity of the sample.

Numerical evaluations of Eq. (1) and a Monte Carlo validation of the above model (see Fig. 11 and the associated text) show that for large *fom* values the cross-correlation distribution is more peaked and with smaller tails. Decreasing the *fom* and keeping all other variables the same, leads to a less peaked distribution with larger tails. The behaviour of the cross-correlation distribution with respect to the mean number of photoelectrons created in the PMTs can be explained intuitively in the following way: suppose that there are overall *n* detected photons of which roughly *n*/2 will be detected by each PMT. Consider the case with a large *n* and many photons detected in both PMTs. The probability to have a small time difference between the first photons arriving in each PMT will be larger compared to a situation with a small number of detected photons. In the latter case the variations of the arrival times of the first photons in the PMTs will be larger and the cross-correlation distribution less peaked and with wider tails. The effect is purely statistical and arises from the dependence of the variation of the difference in arrival times on the number of detected photons per decay.

### Measurement of cross-correlation spectra

In order to study the properties of the cross-correlation distribution, experimental spectra were acquired using the Compton coincidences method with fast signal digitization and offline data processing. The method allows to produce monoenergetic electrons in a LS sample with energies between 2.5 and 8 keV. The data for offline processing contains all the necessary information about the events detected by each PMT, i.e. time stamp and pulse amplitude, which can be used to evaluate the counting rates *and* the cross-correlation spectrum.

**Figure 2:**
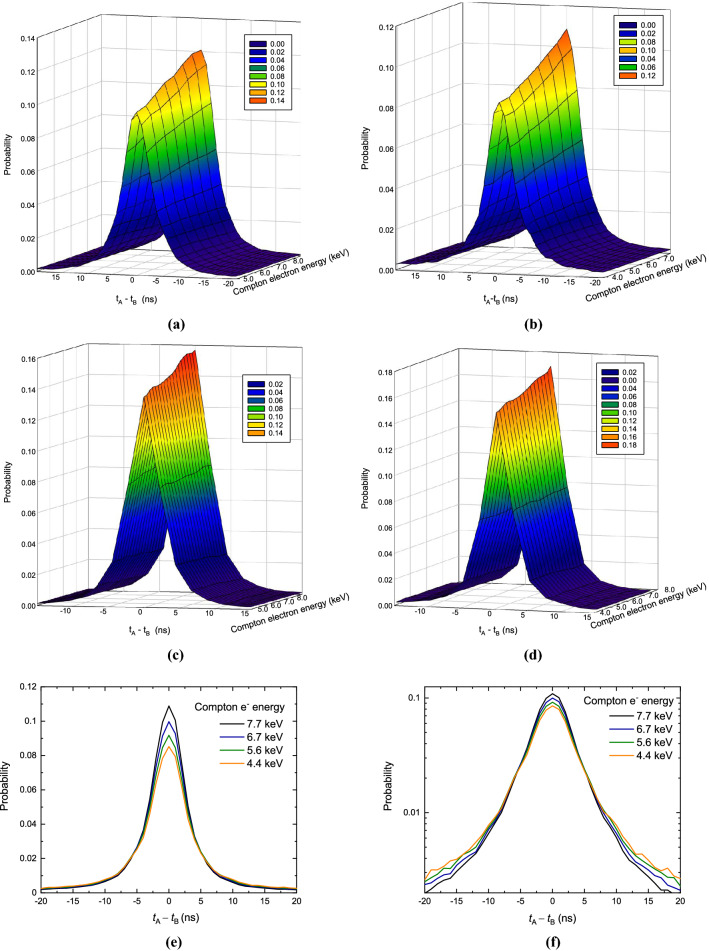
Cross-correlation spectra $D(\Delta t)$ of different LS cocktails acquired by the Compton coincidences method: (**a**) Ultima Gold; (**b**) Ultima Gold LLT; (**c**) Hionic Fluor; (**d**) Toluene, PPO, POPOP locally made cocktail; cross-correlation distribution for several deposited energies measured in Ultima Gold LLT liquid scintillation cocktail in linear (**e**) and log-scale (**f**).

Measurements of four commonly used commercial liquid scintillation cocktails were performed and the results are shown in Fig. 2. The cross-correlation distribution is shown at different energies deposited by Compton electrons in the cocktail. The results in Fig. 2 demonstrate that the larger the deposited energy is, the higher and less tailed the cross-correlation distribution becomes. These effects are also depicted in Fig. 2e,f, where Fig. 2e emphasizes the height dependence on the deposited energy and Fig. 2f focuses on the behaviour of the tails. Note that the average number of detected photons is directly connected to the energy deposited in the cocktail. It follows that the experimental results are in agreement with the prediction of the theoretical model (Eq. 1) and the behaviour observed in the Monte Carlo simulations (Fig. 11). It is clear that the cross-correlation distribution contains information about the mean number of detected photons which allows the calculation of the detection efficiency. The challenging point becomes how this information can be extracted and used.

### Cross-correlations and TDCR counting

The TDCR method allows to estimate the *fom* from a LSC measurement with a three PMT system (see “Methods” below). The cross-correlation approach allows the determination of the same parameter and therefore, it interesting to compare the *fom* values obtained with the two approaches.

A direct way to extract the parameters of the measurement $\varphi$, $\lambda$, $\sigma$ and $\mu$ within the cross-correlation approach is to fit the experimental cross-correlation distribution with the function $D(\Delta t; \varphi , \lambda , \epsilon _A, \epsilon _B, \mu , \sigma )$ given explicitly in Eq. (1). However, the experiments show that the parameters $\varphi$ and $\lambda$ are highly correlated and in order to obtain the correct $\varphi$ it is necessary to fix the correct $\lambda$ or vice versa. Generally, the fast decay constant of the scintillator can be obtained by other methods, for example by time-correlated single photon counting^12^. The parameters concerning the measurement system can be estimated by careful characterization of the time response of PMTs and their relative quantum efficiencies.

The following experiment was undertaken to compare the values of the *fom* obtained by TDCR and cross-correlation methods: a set of measurements of a LSC source are recorded using various grey filters around the source, in order to change the *fom* without modifying the other parameters of the experiment. The measurement data is digitized and saved for offline processing to enable the simultaneous application of the TDCR and cross-correlation methods. Then, the TDCR estimate of $\varphi$ from the measurement without filter is fixed in $D(\Delta t; \varphi , \lambda , \epsilon _A, \epsilon _B, \mu , \sigma )$ and the cross-correlation distribution for the same measurement (i.e. without filter) is fitted to determine $\lambda$. This value of $\lambda$ is used in all subsequent fits of cross-correlation data obtained with filters as the fast decay constant parameter $\lambda$ is a property of the sample and does not depend on the detector geometry and can be considered constant for the cross-correlation measurements of the same LSC source measured with various grey filters.

**Figure 3:**
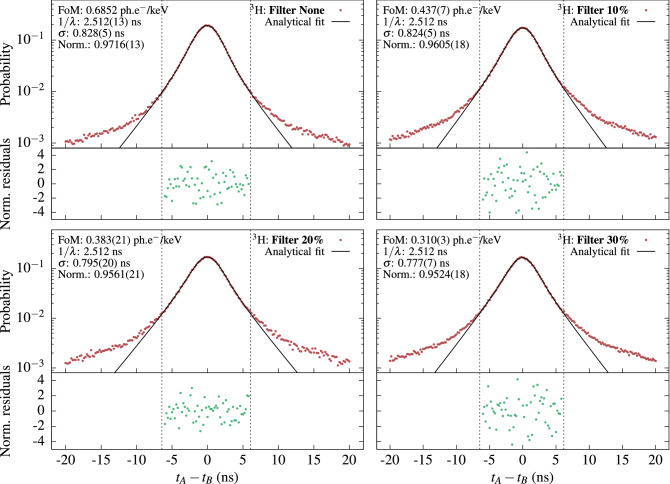
Cross-correlation spectra of a $^3$H sample with varying levels of grey filters. Top left: without filter, top right: filter with 10% opacity, bottom left: filter with 20% opacity, bottom right: filter with 30% opacity. The spectra are fitted with Eq. (29). The normalized residuals are in units of standard deviations. The values in brackets are the uncertainties as reported by the fitting algorithm. The parameters, for which the values are given without uncertainties were fixed during the fitting.

The experiments were performed with $^3$H and $^{14}$C LS-sources. The $^3$H source is measured without filter and with three grey filters with opacity 10%, 20% and 30%, respectively. For both nuclides the measurement without filter was used to determine the decay constant of the scintillator $\lambda$. The obtained cross-correlation spectra and the fits of the $^3$H sample are shown in Fig. 3. The $^{14}$C results and fitting procedures can be found below in “Methods”.

The list-mode files obtained during the $^3$H measurement without filter were analyzed according to the TDCR method and the relative quantum efficiencies ($\varepsilon _A$, $\varepsilon _B$ and $\varepsilon _C$) of the three PMTs were obtained as well as the *fom* $\varphi$, using a dedicated calculation code^13^. The cross-correlation spectrum of PMTs B and C was also obtained. These two PMTs were selected due to their almost identical quantum efficiencies and gain. Note here that the *fom* for a pair of PMTs, for example B and C, is $\varphi _{BC} = \varphi (\varepsilon _B + \varepsilon _C)$, where $\varphi$ is the *fom* from the TDCR measurement. The experimental points were fitted using Eq. (29) by fixing the value of the *fom* to 0.685 ph.e$^-$/keV, as obtained from the TDCR method, and leaving all other parameters free. The fit was performed on data between − 6 and 6 ns as the cross-correlation spectrum seems to be significantly affected by delayed fluorescence for larger time differences. The data and fit are shown in Fig. (3) in the top left sub-figure. The quality of the fit is good as most residuals lie within two standard deviations. Notice that a normalization parameter (*Norm.*) is multiplying Eq. (29) in order to accommodate the fact that not all of the cross-correlation spectrum can be explained by the prompt fluorescence only. From this measurement the prompt fluorescence decay time $\lambda$ of the scintillator was determined to be 2.512(13) ns. This decay time was then used in all subsequent analysis of measurements of the $^3$H sample.

The comparison between the TDCR and cross-correlation estimated *fom* values is shown in Table 1. The *fom* parameters obtained with the two measurement methods agree well within the estimated uncertainties. The results indicate that, with a good knowledge of the prompt fluorescence decay constant, the cross-correlation method provides reliable estimation of the *fom*.

**Table 1:** Comparison of the *fom* obtained by cross-correlation and TDCR measurements with various levels of grey filters for $^3$H and $^{14}$C LSC sources.

$^3\hbox {H}$: *fom* $\varphi$, ph.e$^-/\hbox {keV}$			$^{14}\hbox {C}$: *fom* $\varphi$, $\hbox {ph.e}^-/\hbox {keV}$		
Filter	Cross-corr.	TDCR	Filter	Cross-corr.	TDCR
None	–	0.685(20)	None	–	0.326(6)
90%	0.437(7)	0.434(13)	80%	0.254(6)	0.267(4)
80%	0.383(21)	0.370(11)	70%	0.229(9)	0.233(4)
70%	0.310(3)	0.312(9)	60%	0.201(8)	0.191(3)

### Height and Kurtosis of the cross-correlation spectrum

**Figure 4:**
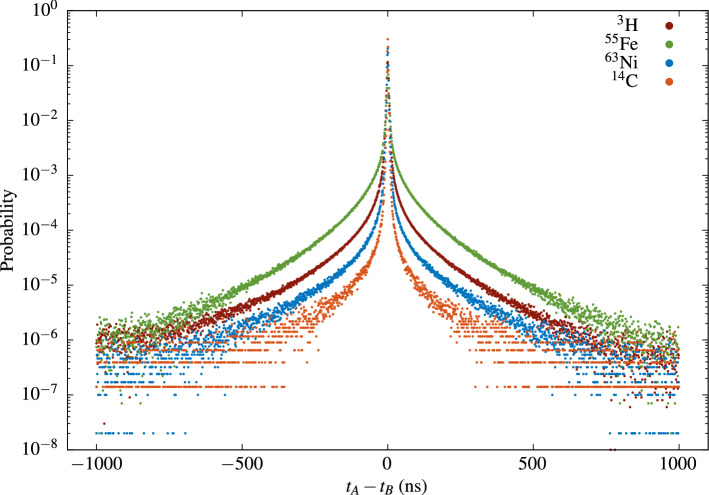
Cross-correlation spectra of $^{55}$Fe, $^3$H, $^{14}$C and $^{63}$Ni.

Cross-correlation spectra of $^{55}$Fe ($\hbox {E}_{\text{mean}}$ $\approx$ 5.5 keV), $^3$H ($\hbox {E}_{\text{mean}} = 5.68~\hbox {keV}$), $^{14}$C ($\hbox {E}_{\text{mean}} = 49.16~\hbox {keV}$) and $^{63}$Ni ($\hbox {E}_{\text{mean}}=17.43~\hbox {keV}$) sources, prepared in Ultima Gold LS cocktail using diffusive (sandblasted) glass vials, were acquired. The cross-correlation was measured between two PMTs and the detection efficiency variation was achieved by means of grey filters. Figure 4 depicts the cross-correlation distributions of all samples without filters. These distributions are obtained by normalization of the measured cross-correlation spectra on the total number of events in the spectrum. The results in the figure confirm the aforementioned theoretical and experimental findings that higher energy deposited in the cocktail leads to more peaked and less tailed cross-correlation distribution. This was previously observed by other authors^14^.

The maximum of the cross-correlation distribution is located at $\Delta t = \mu$. Note that for PMTs with the same static time delay $\mu$ equals 0 and the maximum is located at $\Delta t=0$. Thus, the peak height $H_0$ of the cross-correlation distribution is given by:

10 $$\begin{aligned} H_0= D(\Delta t= \mu ; \varphi , \lambda , \epsilon _A, \epsilon _B, \mu , \sigma ) \end{aligned}$$

**Figure 5:**
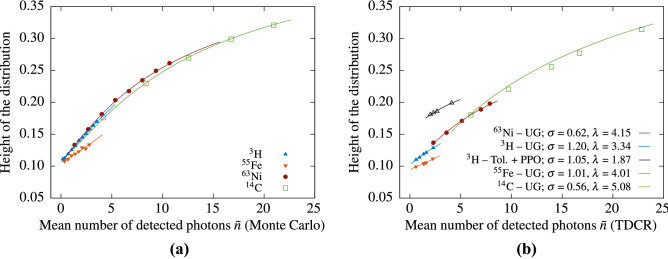
Height of the normalized cross-correlation spectra as a function of the mean number of detected photons. The data points on the left are generated by the Monte Carlo code and the lines are calculated from the analytical equation with the same parameters at $\Delta t = \mu$. The data points on the right are from TDCR measurements of $^3$H, $^{55}$Fe, $^{64}$Ni and $^{14}$C in Ultima Gold and $^3$H in toluene + PPO cocktails. The fit is done with the analytical equation at $\Delta t = \mu$ in order to obtain the optimal $\lambda$ and $\sigma$ parameters.

The experimental results show that, for fixed $\lambda$, $\mu$ and $\sigma$ and for small average number of photons ${\bar{n}}$ the height of the cross-correlation distribution $H_0$, depends almost linearly on ${\bar{n}}$. For large ${\bar{n}}$ the response function of the detector leads to a non-linear behaviour. The actual dependence can be found by fixing $\Delta t = \mu$ in Eq. (29) and varying the parameters $\lambda$ and $\sigma$. This is illustrated in Fig. 5a for a set of Monte Carlo generated time distributions, where the lines are evaluated using the analytical equation with the same $\lambda$, $\sigma$, $\mu$ and $\epsilon$ parameters as in the input of the Monte Carlo code. The height of the distribution as a function of the average number of photons was also obtained for measurements of the LSC sources with and without grey filters. The relationships for each nuclide were fitted with the analytical Eq. (29) for a fixed $\Delta t = \mu$. The quantum efficiencies of the PMTs are assumed to be identical and the $\mu$ parameter is calculated as the mean of the distributions. The two other free parameters, the $\lambda$ and $\sigma$ were varied until a satisfactory fit was achieved. The obtained parameters and fitted curves are shown in Fig. 5b. These results show that, for a given radionuclide, knowing the decay constant of the scintillator $\lambda$ and the parameters of the detection system ($\mu$, $\sigma$), it is possible to obtain the average number of detected photons ${\bar{n}}$ and thus the detection efficiency of the measurement by measuring the height of the cross correlation distribution.

Similar to the height, the tails of the distribution also depend on the *fom*. It is thus pertinent to consider the fourth moment of the cross-correlation distribution, the Kurtosis *K*, in order to characterize them:

11 $$\begin{aligned} K=\mathop {{\mathbb {E}}} \left[ \left( \frac{\Delta t-\overline{\Delta t}}{\sigma _D} \right) ^4\right] \end{aligned}$$

where $\overline{\Delta t}$ and $\sigma _D$ are the mean and the standard deviation of the cross-correlation distribution and ${\mathbb {E}}$ stands for the expected value.

**Figure 6:**
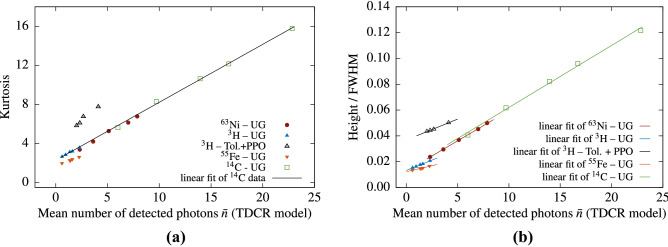
Kurtosis of the cross-correlation spectra—the data points are measurement results and the linear fit is performed on the $^{14}$C data and extrapolated (**a**). The average number of detected photons is calculated from the TDCR model. (**b**) Scaling the cross-correlation data $H_0$/FWHM vs ${{\bar{n}}}$. The data points are from measurements of various LS-sources and the average number of photons is calculated from the TDCR model.

Good linear relations are observed between the Kurtosis *K* and the average number of detected photons (Fig.  6). Figure 6a depicts *K* vs. ${{\bar{n}}}$ results obtained in measurements with a three PMT system. The mean number of detected photons in this case is calculated from the TDCR model. Note that, the experimental points for *K* vs ${\bar{n}}$ seem to belong to the same line for all beta-emitting nuclides in UG cocktail except for $^{55}$Fe (Fig. 6a). We found empirically that similar common scaling can also be obtained with $H_0$ if we consider $\frac{H_0}{\text {FWHM}}$ vs ${{\bar{n}}}$ (Fig. 6b), where FWHM stays for the full with at half maximum of the cross-correlation distribution.

Thus, considering the above results it can be speculated that, in a given LSC system and a given cocktail, the cross correlation data for low energy beta emitters like $^3$H, $^{14}$C and $^{63}$Ni may be presented as (or scaled to) a linear function of the mean number of detected photons during the measurement. The data for the electron capture nuclide $^{55}$Fe also shows a linear behaviour, but does not scale to the same linear dependence as that for the low-energy beta-emitters.

### Cross-correlations and conventional liquid scintillation analysis

In order to explore the application of cross-correlations to the problem of efficiency estimation in routine LS analysis, we searched for a connection between the parameters describing the cross-correlation and those describing the efficiency of the sample measurement. It should be noted from the outset that, according to Eq. (7) there is a unique relation between the efficiency of a LSC system and the *fom*. This relation is the basis of the free-parameter model^8^ used in the CNET and TDCR activity standardization techniques.

**Figure 7:**
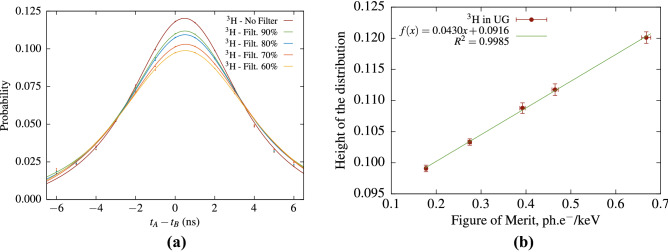
Cross-correlation studies with $^3$H in Ultima Gold cocktail and various levels of grey filters. The cross-correlation spectra (**a**) are fitted within ± 3 ns with a Voigt profile in order to evaluate the height of the distribution.

**Figure 8:**
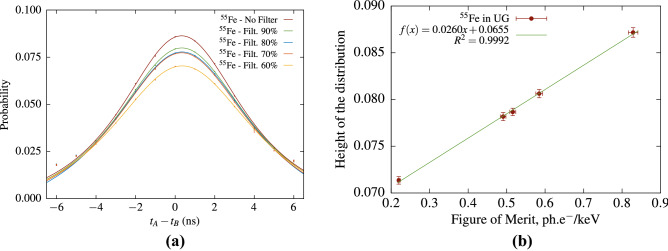
Cross-correlation studies with $^{55}$Fe in Ultima Gold cocktail and various levels of grey filters. The cross-correlation spectra (**a**) are fitted within ± 3 ns with a Voigt profile in order to evaluate the height of the distribution.

**Figure 9:**
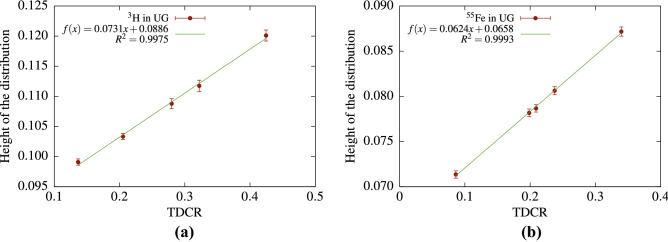
Height of the cross-correlation distribution ($H_0$) vs *TDCR* value for $^3$H (**a**) and $^{55}$Fe (**b**) sources in Ultima Gold cocktail.

For the purpose of routine LS analysis it seems meaningful to abandon for a moment the complex theoretical cross-correlation equations (e.g., Eq. 29 or Eq. 31) and to consider an empirical characterization of the cross-correlation spectra. Figure 7 shows the cross-correlation distribution in the $^3$H case. In order to simplify the data analysis and highlight the physical aspects, the height of the distribution $H_0$ is determined by fitting the cross-correlation data in the range [− 3 ns, 3 ns] with a Voigt profile. The uncertainty of $H_0$ is obtained from the covariance matrix reported by the fitting algorithm and is in the order of 0.7%. As the *fom* is measured by means of the TDCR method and the free-parameter model it also has an associated uncertainty, which was estimated by varying the model parameters. The error bars in figures Figs. 7b and 9 show the standard estimates uncertainties. Similar results were obtained in the case of $^{55}$Fe (Fig. 8). Figures 7b and 8b show that there is an excellent linear relation between $H_0$ and the *fom*.

A commonly used approach in routine liquid scintillation analysis is to determine the detection efficiency from a quenching indicator using predetermined quench curves. There are many quenching indicators depending on the manufacturer of the LSC analyzer, but they all rely on the analysis of the pulse-height spectrum of the sample acquired with an external gamma source. A recent study^15^ has shown that the TDCR value can also be used as a quench indicating parameter. Here we present results of TDCR measurements of $^3$H and $^{55}$Fe in Ultima Gold cocktail with efficiency variation performed by means of grey filters. The results indicate that there is a linear relation between the height of the cross-correlation distribution $H_0$ (Eq. 10) and the measured TDCR value in a very large interval (Fig. 9). The uncertainty of $H_0$ is the same as in Figs. 7 and 8.

Consequently, it appears that the parameters characterizing the cross-correlation distribution may serve as efficiency indicators in conventional LS counting measurements.

## Discussion

The main result from this work is the fact that the time domain and particularly the cross-correlation distribution contain information about the detection efficiency of a LSC measurement. The presented theoretical and experimental results demonstrate that the analysis of the cross-correlation distribution can provide very useful information for the other LSC methods. This includes, for instance, the assessment of the quenching parameter for activity standardization by the CNET method and for checking the consistency of the TDCR method. Another example is the proposition of new efficiency indicators to be used in routine LS analysis like the height or the kurtosis of the cross-correlation distribution. The cross-correlation distribution relies on time-domain data and thus the information obtained with it cannot be considered equivalent to the one obtained by the commonly used LSC methods. In this regard the cross-correlation analysis can be regarded as an extra method to be used with or within the other LS techniques.

A main advantage of the newly proposed method is that the cross-correlation information can be derived directly from the measurement itself using an ad-hoc acquisition system. A typical example is the usage of fast digitizers to record the measurement data in a list-mode format (e.g., event timestamp, event information). In this case, the LSC data (counting rates) and the cross-correlation distribution (efficiency information) can be extracted from the same list-mode file. Thus, the variability due to differences between measurement and calibration conditions is largely minimized. The use of the cross-correlation distribution suggests also that a new type of LSC systems can be developed: it seems feasible to develop electronics circuits for simultaneous acquisition of coincidence counting rate and cross-correlation data. LSC analyzers with such electronics should be able to determine the activity of a sample with given liquid scintillator characteristics from a single measurement by simultaneous estimation of counting rate and efficiency. They will also avoid the need for external radioactive sources for the determination of quenching indicators.

There are several challenges in front of the application of the cross-correlation method. From the point of view of primary metrology (activity standardization by LSC) the difficulties come from the liquid scintillation cocktails. Ideally, liquid scintillation cocktails with short rise time and separated or suppressed/inhibited delayed component should be used. Such cocktails will suppress or even eliminate the influence of the delayed scintillation component on the peak region of the cross-correlation spectrum. For them it will be easier to maintain the assumptions for which Eq. (1) is derived. The difficulties caused by the delayed scintillation component are not specific to this type of measurement method, as it also causes problems in TDCR counting. These are mainly due to differences in the ionization quenching behavior between the prompt and delayed scintillation components^16^. Therefore, despite the long history of the field, it is still worth putting efforts in the development of a stable LSC cocktail with: known material content, high light output, ideally with a fast and isolated prompt component and suppressed delayed scintillation component. Such a cocktail will be useful for all the methods applying LSC for activity standardization.

From experimental point of view, high-quality PMTs have to be used for cross-correlation measurements. This means high quantum efficiency, single photon sensitive PMTs with a single electron peak that is well separated from the noise. In addition, matched PMTs with similar and fast response as well as with small transient time spread will provide optimal timing performance. Fast digitizers with constant fraction discrimination or fast circuits with timing resolution better than 200 ps are required. The time-response properties of the system, described by $\lambda$, $\mu$ and $\sigma$ in the cross-correlation distribution $D(\Delta t; \varphi , \lambda , \epsilon _A, \epsilon _B, \mu , \sigma )$, have to be carefully characterized. The parameters $\mu$ and $\sigma$ are specific to the detector system and depend on the PMTs and the associated electronics. The decay constant $\lambda$ is a property of the LSC sample. Unfortunately, the decay constant $\lambda$ and the figure of merit $\varphi$ cannot be determined from a single fit of the cross-correlation distribution because they are strongly correlated. The solution is to use additional measurements as it is done in this work or, alternatively, to use other techniques, such as time-correlated single photon counting, to the determine the decay constant $\lambda$. The latter option is very promising as it allows completely independent determination of $\lambda$ for a particular LSC sample and thus will largely facilitate the determination of the *fom*. This approach will be explored in future studies.

Thus far, the cross-correlation method has been tested experimentally with low-energy beta-emitters and electron-capture radionuclides. Experiments with high-energy beta-emitters or alpha- emitting radionuclides have not been performed yet. However, based on our current experience, we do not see any principle obstacle to the application of the cross-correlation method in these cases. According to our current understanding, the cross-correlation method cannot be used for discrimination between alpha- and beta-pulses. In the case of counting samples containing alpha- and beta-emitters, it can probably be applied on the alpha- or beta-data, obtained after the application of traditional pulse-shape discrimination techniques. All these applications need to be tested in further studies. Finally, a full uncertainty budget of the detection efficiency derived from the cross-correlation method should be established for the application of this method in radionuclide metrology.

## Conclusions

In this work we stress that the time domain in LSC, and the cross-correlation distribution in particular, contain important information about the detection efficiency. It is demonstrated how the cross-correlation data can be used to estimate the figure of merit of the measurement, which gives the detection efficiency. Overall, the concept presented in this work can bring improvements in almost all applications of LSC to activity determination of low-energy beta-emitting and electron-capture radionuclides. The results are also promising for the development of innovative, simpler, more functional and in the same time better performing LSC systems. Research is ongoing in order to reveal the full potential of the cross-correlation method.

## Methods

### Derivation of time distribution between PMT events

Let us consider the case where an electron with energy *E* is absorbed by the scintillator and produces *n* excited states $x_1, x_2,\dots , x_n$ that will all emit photons that will be detected. Let, for each state, that the probability ($p_i$) to de-excite at a given time *t* is an exponential distribution with a decay constant $\lambda$:

12 $$\begin{aligned} p_i(t) = \lambda e^{-\lambda t}. \end{aligned}$$

We are concerned with the distribution of the time of emission from the state that de-excites first, as that state will form the rising edge of the detector signal and will serve as the start or stop in the timing circuit. The probability for the state $x_1$ to be the first de-exciting is the probability of $x_1$ to de-excite at moment $t$ and all the other states to de-excite after it:

13 $$\begin{aligned} p_{x1} (t) = \lambda e^{-\lambda t} \left[ \int _t^\infty \lambda e^{-\lambda t} dt\right] ^{n-1} = \lambda e^{-n\lambda t} \end{aligned}$$

where the second term is the probability of all other states to de-excite after $x_1$. The probability of the first photon arriving at time $t$ from any of the *n* states is then:

14 $$\begin{aligned} P_i(t) = p_{x1} + p_{x2} +\cdots + p_{xn} = n\lambda e^{-n\lambda t}, \end{aligned}$$

where *i* denotes one of the PMTs.

For a detector system with two PMTs the total number of detected photons *n* will be the sum of *k* detected in the one PMT and $n-k$ detected in the other. If we denote the two PMTs with A and B, and if we assume that the first emitted photon will be the first to be detected, then according to Eq. (14) the probability for detection of the first photon at a time $t_A$ in PMT A or $t_B$ in PMT B will be:

15 $$\begin{aligned}&P_A(t_A) = k\lambda e^{-k\lambda t_A} \\& P_B(t_B) = (n-k)\lambda e^{-(n-k)\lambda t_B} \end{aligned}$$

We assume also that the time response of the PMTs is described by a Gaussian function of the type $G(t;\mu _i,\sigma _i)={\frac{1}{\sqrt{2\pi }\sigma _i}}exp\left( -\frac{(t-\mu _i)^2}{2{\sigma _i}^2}\right)$, where $i = {A, B}$ for the two PMTs. Then the probability $PG_i$ to obtain a pulse which detects the first event in one of the PMT becomes:

16 $$\begin{aligned} \begin{aligned} PG_A(t_A; n, k, \lambda , \mu _A, \sigma _A)=P_A(t_A; n, k, \lambda ){*}G_A(t; \mu _A, \sigma _A) \\ PG_B(t_B; n, k, \lambda , \mu _A, \sigma _A)=P_B(t_B; n, k, \lambda ){*}G_B(t; \mu _B, \sigma _B) \end{aligned} \end{aligned}$$

where $*$ indicates convolution.

We will be interested in the probability to obtain a time difference $\Delta t_{AB}=t_A-t_B$ between the pulses from PMTs A and B, which is given by the cross-correlation of $PG_A(t)$ and $PG_B(t)$. Similarly, the time differences between B and A $\Delta t_{BA} = t_B - t_A$ is given by the cross-correlation of $PG_B(t)$ and $PG_A(t)$.

17 $$\begin{aligned} \begin{aligned} PG_{AB}({\Delta t})=PG_A(t_A)\star PG_B(t_B),\\ PG_{BA}({\Delta t})=PG_B(t_B)\star PG_A(t_A), \end{aligned} \end{aligned}$$

where $\star$ denotes the cross-correlation operator. The sum of the distributions $PG_{AB}$ and $PG_{BA}$ will be referred to as the cross-correlation distribution between PMTs A and B. Noting that the cross-correlation of functions *f*(*t*) and *g*(*t*) is equivalent to the convolution of *f*(*t*) with $g(-t)$ one gets also:

18 $$\begin{aligned} \begin{aligned} PG_{AB}(\Delta t)= PG_A(t_A)*PG_B(-t_B),\\ PG_{BA}(\Delta t) = PG_B(t_B)*PG_A(-t_A). \end{aligned} \end{aligned}$$

Substituting Eq. (16) in (18) and using the associativity of the convolution operator one obtains:

19 $$\begin{aligned} \begin{aligned} PG_{AB}(\Delta t_{AB})&=(P_A(t_A)*P_B(-t_B))*(G_A(t_A)*G_B(-t_B)),\\ PG_{BA}(\Delta t_{BA})&=(P_B(t_B)*P_A(-t_A))*(G_A(t_B)*G_A(-t_A)). \end{aligned} \end{aligned}$$

In order to simplify the notation, it is useful to define $\Delta t = \Delta t_{AB} = -\Delta t_{BA}$. Explicitly, the distribution $P_{AB}(\Delta t; n, k, \lambda )=P_A(t_A)*P_B(-t_B)$ is given by:

20 $$\begin{aligned} P_{AB}(\Delta t; n, k, \lambda )= {\left\{ \begin{array}{ll} \frac{k(n-k)}{n}\lambda e^{-(n-k)\lambda \Delta t}, &{}\text {for}\; \Delta t \ge 0,\\ 0, &{}\text {for}\; \Delta t \le 0. \end{array}\right. } \end{aligned}$$

Similarly, for $$P_{BA}(\Delta t; n, k, \lambda ) = P_B(t_B)*P_A(-t_A)$$:

21 $$\begin{aligned} P_{BA}(\Delta t; n, k, \lambda )= {\left\{ \begin{array}{ll} 0, &{}\text {for }\Delta t \ge 0,\\ \frac{k(n-k)}{n}\lambda e^{-k\lambda \Delta t}, &{}\text {for }\Delta t \le 0. \end{array}\right. } \end{aligned}$$

Noting that the convolution of two Gaussian distributions is a Gaussian distribution and that $G(-t; \mu , \sigma ) = G(t; -\mu , \sigma$) one gets:

22 $$\begin{aligned}  &G_{AB}(\Delta t; \mu , \sigma ) = G_A(t_A; \mu _A, \sigma _A) *G_B(t_B; -\mu _B, \sigma _B),\\&G_{BA}(\Delta t; \mu , \sigma ) = G_B(t_B; -\mu _B, \sigma _B) *G_A(t_A; \mu _A, \sigma _A), \end{aligned} $$

with $$\mu = \mu _A - \mu _B$$ and $$\sigma ^2 = \sigma _A^2 + \sigma _B^2$$.

Finally, for the cross-correlation distributions $PG_{AB}(t)$ and $PG_{BA}(t)$, one obtains the following equations:

23 $$\begin{aligned}&PG_{AB}(\Delta t; n, k, \lambda , \mu , \sigma )= P_{AB}(\Delta t; n, k, \lambda ) *G_{AB}(\Delta t; \mu , \sigma ) \end{aligned}$$

24 $$\begin{aligned}&PG_{BA}(\Delta t; n, k, \lambda , \mu , \sigma ) = P_{BA}(\Delta t; n, k, \lambda ) *G_{BA}(\Delta t; -\mu , \sigma ), \end{aligned}$$

The convolution of an exponential distribution of the type $f(x) = \tau e^{-\tau x}$ and a Gaussian distribution is the exponentially modified Gaussian distribution with the form:

25 $$\begin{aligned} EMG(t; \tau , \mu , \sigma ) = \frac{\tau }{2} e^{\frac{\tau }{2} \left( 2\mu + \tau \sigma ^2 - 2t\right) } \text {erfc}\left( \frac{\mu + \tau \sigma ^2 - t}{\sqrt{2}\sigma } \right) , \end{aligned}$$

where erfc denotes the complementary error function $\text {erfc}(x) = 1 - \text {erf}(x)$. In order to evaluate the convolutions in (23) and (24), the following substitutions can be made: $t = \Delta t$ and $\tau = k\lambda$ in the case of $PG_{AB}$ and $t = -\Delta t$ and $\tau = (n-k)\lambda$ in the case of $PG_{BA}$. The total distribution of the time intervals $\Delta t$ then becomes:

26 $$\begin{aligned} A(\Delta t; n, k, \lambda , \mu , \sigma ) = \frac{1}{n} \Bigl ( k~EMG_{AB}\left( \Delta t; (n-k)\lambda , \mu , \sigma \right) + (n-k)~EMG_{BA}\left( -\Delta t; k\lambda , -\mu , \sigma \right) \Bigr ). \end{aligned}$$

The probability to detect exactly *k* photons in one PMT and $m = n - k$ in the other out of *n* detected photons per ionizing particle is given by the binomial distribution. Thus, summing over all possible *n* and *k* pairs one obtains the cross-correlation distribution between two PMTs for a fixed number of exactly *n* detected photons:

27 $$\begin{aligned} B(\Delta t; n, \lambda , \epsilon _A, \epsilon _B, \mu , \sigma ) = \sum _{k = 1}^{n-1} \left( {\begin{array}{c}n\\ k\end{array}}\right) \epsilon ^k_A\epsilon ^{n-k}_B~A(\Delta t; n, k, \lambda , \mu , \sigma ), \end{aligned}$$

where $\epsilon _A$ and $\epsilon _B$ are the relative efficiencies of the PMTs which satisfy the equality $\epsilon _A+\epsilon _B=1$. It should be noted that the binomial coefficients are summed from 1 to $n - 1$, because events with zero hits in either PMT will not result in a detected coincidence. In a real situation the number of excited states for a deposited energy *E* will not be a fixed number *n*, but will follow a Poisson distribution with a mean number ${\bar{n}}$. By summing through all possible *n* we obtain:

28 $$\begin{aligned} C(\Delta t; {\bar{n}}, \lambda , \epsilon _A, \epsilon _B, \mu , \sigma ) = \sum _2^\infty \sum _{k = 1}^{n-1} \frac{{\bar{n}}^n}{n!} e^{-{\bar{n}}} \left( {\begin{array}{c}n\\ k\end{array}}\right) \epsilon ^k_A\epsilon ^{n-k}_B ~A(\Delta t; n, k, \lambda , \mu , \sigma ), \end{aligned}$$

Equation (28) is sufficient to describe the cross correlation in the case of mono-energetic electrons. If the source is a $\beta$-emitting radionuclide, then its $\beta$-spectrum must be taken into account. Taking into account the spectrum of the deposited energy in the scintillator *S*(*E*) and the relationship between ${\bar{n}}$ and the deposited energy given by Eq. (3), the cross-correlation becomes:

29 $$\begin{aligned} D(\Delta t; \varphi , \lambda , \epsilon _A, \epsilon _B, \mu , \sigma ) = \frac{1}{L} \int _0^{\text {E}_\text {{max}}} S(E) \sum _2^\infty \frac{\left( {\bar{n}}(E; \varphi )\right) ^n}{n!} e^{-{\bar{n}}(E; \varphi )} \sum _{k = 1}^{n-1} \left( {\begin{array}{c}n\\ k\end{array}}\right) \epsilon ^k_A\epsilon ^{n-k}_B ~A (\Delta t; n, k, \lambda , \mu , \sigma )~dE, \end{aligned}$$

where $E_\text {max}$ is the maximum energy in the particle spectrum and *L* is a normalization coefficient equal to the probability of all detected events:

30 $$\begin{aligned} L = \int _0^{\text {E}_\text {{max}}} S(E) \sum _2^\infty \frac{\left( {\bar{n}}(E; \varphi )\right) ^n}{n!} e^{-{\bar{n}}(E; \varphi )} \sum _{k = 1}^{n-1} \left( {\begin{array}{c}n\\ k\end{array}}\right) \epsilon ^k_A\epsilon ^{n-k}_B~dE. \end{aligned}$$

The normalization is needed because the events with less than one detected photon per PMT would not make coincidences and will not be detected by the system.

Equations (29) and (30) are for two PMT counting systems. Similar equations can be derived also for three PMT systems, considering that probability to have *k* photons in PMT A, *m* photons in PMT B and $l=n-k-m$ in PMT C can be calculated by the multinomial distribution. Thus, the equivalent of Eq. (29) for a three PMT system is:

31 $$\begin{aligned} D'(\Delta t; \varphi , \lambda , \varepsilon , \mu , \sigma ) = \frac{1}{L'} \int _0^{\text {E}_\text {{max}}} S(E) \sum _2^\infty \frac{\left( {\bar{n}}(E; \varphi )\right) ^n}{n!} e^{-{\bar{n}}(E; \varphi )} \sum _{k = 1}^{n-1}\sum _{m = 1}^{n-k} \frac{n!}{k!m!l!} \epsilon ^k_A\epsilon ^m_B\epsilon _C^{l} ~A (\Delta t; k, m, \lambda , \mu , \sigma )~dE, \end{aligned}$$

where $\epsilon _A$, $\epsilon _B$ and $\epsilon _C$ are the measured relative efficiencies of the PMTs and $L'$ is the normalization constant given by:

32 $$\begin{aligned} L' = \int _0^{\text {E}_\text {{max}}} S(E) \sum _2^\infty \frac{\left( {\bar{n}}(E; \varphi )\right) ^n}{n!} e^{-{\bar{n}}(E; \varphi )} \sum _{k = 1}^{n-1}\sum _{m = 1}^{n-k} \frac{n!}{k!m!l!} \epsilon ^k_A\epsilon ^m_B\epsilon _C^l~dE \end{aligned}$$

### TDCR method

The TDCR method is widely used by national metrology institutes for primary standardization of LSC sources^2^. It is especially suited for the determination of the activity of pure beta and electron capture radionuclides. The method is based on a model that provides a statistical description of the physical phenomena occurring in the LSC system. The principle and development of the model is summarized in the work of Broda^17^. With the TDCR method one can obtain the detection efficiency of the detector from the ratio of the triple to double coincidences. The application of the method requires the use of a specialized counter with three PMTs and electronics that is able to record the triple (T) and double (AB, BC, AC) coincidences counting rates. A logical sum of double coincidences (D) channel can be defined as the logical *or* of the three double channels. Under the assumption of three identical PMTs the ratio of the detection efficiency in the T channel to that in the D channel is^2^:

33 $$\begin{aligned} \frac{\Phi _\text {T}}{\Phi _\text {D}} = \frac{\displaystyle \int _0^{E_\text {max}} S(E) \left( 1-e^{-{\bar{n}}(E;\varphi )/3}\right) ^3dE}{ \displaystyle \int _0^{E_\text {max}} S(E) \left[ 3\left( 1-e^{-{\bar{n}}(E;\varphi )/3}\right) ^2 - 2\left( 1-e^{-{\bar{n}}(E;\varphi )/3}\right) ^3\right] dE}, \end{aligned}$$

where ${\bar{n}}$ is the average number of photons detected for effective energy *E* deposited in the cocktail and is the same parameter defined in Eq. (3). Note that in this case the number of PMTs is equal to 3 and thus the factor in the denominator in the argument of the exponent. For a large number of detected events the ratio of the T to D coincidences tends towards the ratio of the detection efficiencies or $\text {T}/\text {D} = \Phi _\text {T}/\Phi _\text {D}$. The free parameter $\varphi$ can then be obtained by minimizing the squared difference between the two ratios. In the case of non-identical PMTs a set of three equations must be used, including the relative efficiencies of the three PMTs. The equations are used to optimize the three free parameters of the system $\varphi _A = \varepsilon _A \varphi$, $\varphi _B = \varepsilon _B \varphi$ and $\varphi _C = \varepsilon _C \varphi$.

Due to the specific acquisition electronics needed to employ the TDCR method, it is common to use in-house built detector systems. For the purpose of this study, we used a miniature portable TDCR detector, which was designed and developed at the LNE-LNHB and operated at Sofia University. It has three Hamamatsu R7600-200 square form factor PMTs^18^ placed in a 3D printed housing and optical chamber. The outputs of the PMTs are connected to a CAEN DT5751 digitizer^19^ with 1 ns timing resolution, but 200 ps time resolution can be reached using the digital constant fraction discriminator provided in the firmware of the digitizer.

**Figure 10:**
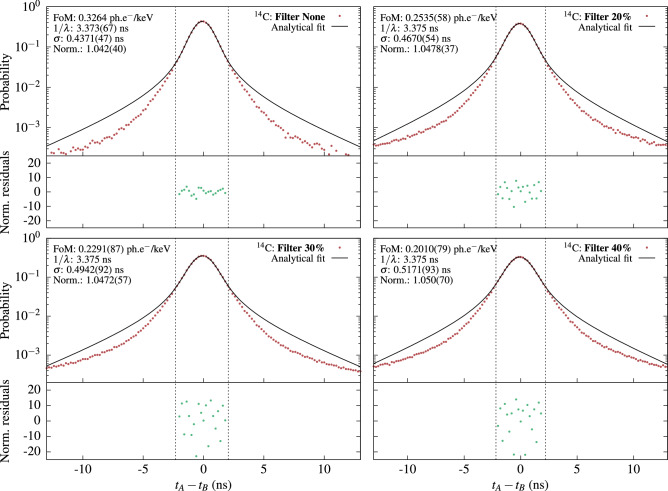
Cross-correlation spectra of a $^{14}$C LS-source in toluene+PPO cocktail. All experimental spectra are fitted with Eq. (29) with a fixed $\lambda$ and normalization parameters.

This detector system can be used for measurements of LS-samples by both the TDCR and cross-correlation methods. A significant advantage is that this allows the direct comparison of the two methods as the figure of merit and PMT efficiencies obtained by the two should be exactly the same. With this system we performed measurements of $^3$H and $^{14}$C LS-samples in a toluene+PPO scintillator. The $^3$H results were shown above in “Results”. The $^{14}$C sample was measured without and with grey filters with opacities 20%, 30% and 40%. The same approach as in the $^3$H measurement was applied. The *fom* of the measurement without a filter was determined from the analysis of the data according to the TDCR method. The value was then used to determine the decay constant $\lambda$ for this cocktail and the rest of the cross-correlation spectra were fitted with a fixed $\lambda$. The *fom* was left as a free parameter. The results are shown in Fig. 10.

Due to the much larger average number of photons in the case of $^{14}$C compared to $^3$H (see Fig. 3) , the cross-correlation distribution is significantly narrower—67% of all events in the distribution fall in the interval between − 1 and 1 ns and 91% fall within − 2 and 2 ns. For $^3$H the intervals are − 2.5 to 2.5 and − 5 to 5 for 67% and 91% respectively. The range that can be successfully fitted by Eq. (29) was found to be ± 2 ns. The events outside this range seem to be significantly affected by the delayed fluorescence. The dispersion of the residuals seems to be considerably larger than for the $^3$H measurements. This could be due to the much narrower time distribution for which minor non-linearities in the bin widths could play a role. Such effects are not included in the uncertainty of the value in each bin which is estimated as the square root of the number of events in the bin.

The TDCR method relies on the accurate description of the relative light output of the scintillator with respect to the deposited energy. This is commonly done with Birks’ ionization quenching formula (see Eq.  4). As the method uses the triple and double coincidence counting rates to calculate the efficiency it is necessary to select coincidence windows that are wide enough to include all correlated events, otherwise a bias may be introduced in the measurement. For too short coincidence windows the loss of triple coincidences will be higher than the loss of double coincidences and an biased efficiency will be obtained. Increased coincidence resolving time would increase the contribution of delayed fluorescence to the overall scintillation light, thus the simple ionization quenching formula proposed by Birks to describe the ionization quenching of prompt fluorescence cannot be used. These issues of the TDCR method were discussed in length by Dutsov et al.^16^.

### Compton coincidences method

The Compton coincidences method was initially developed by Péron and Cassette^20^ and later expanded by Bignell et al.^21^ in order to circumvent the usage of semi-empirical equation that describes the light output of the scintillator. The method uses a three PMT TDCR counter and a gamma-ray detector connected in coincidence. A collimated external source of mono-energetic gamma-rays is placed such that the photon beam passes through the LSC-vial containing the scintillator that is studied. Most of the gamma-rays undergo Compton scattering and produce a Compton electron inside the cocktail. The scattered gamma-ray can interact with the gamma detector and knowing its energy it is possible to calculate the energy deposited in the cocktail by the Compton electron from the energy conservation law. The type of coincidence (double or triple) in the TDCR detector produced by each electron is also recorded. The light output of the scintillator can be obtained from the triple to double coincidences ratio analytically under an assumption for a mono-energetic source^22^. Thus, the relative light output of the scintillator can be obtained as a function of the energy deposited by Compton electrons.

In the current study the Compton coincidences detector that was used was developed at LNE-LNHB. The detector consists of three Hamamatsu R7600-200 square form factor PMTs^18^ and a Cadmium Telluride (CdTe) gamma-ray detector. The PMTs are placed in a 3D printed housing which hosts an optical chamber that is optimized for light collection and is covered with reflective foil with 98% reflectivity in the visible spectrum. The outputs of the PMTs are connected to a CAEN DT5751 digitizer^19^ with 1 ns timing resolution. An external 77 MBq $^{241}$Am source was used as a mono-energetic source of 59.54 keV gamma-rays. The source was filtered in order to remove the lower energy X-rays of $^{237}$Np. This setup allows to study the response of the scintillator to electrons with energies from 2.5 to 8.5 keV in 270 eV steps. The outputs of the three PMTs and the CdTe detector were all connected to the same digitizer mentioned above. The timestamps and energies of each event were recorded in list-mode files and were analyzed offline to obtain the cross-correlation distributions for a number of deposited energies in the scintillator.

### Monte Carlo simulation of time distributions

**Figure 11:**
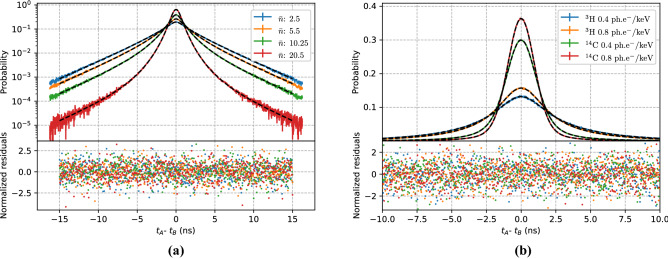
Left: Monte Carlo simulation of time distributions for various average number of photons ${\bar{n}}$ detected by two PMTs and the time distributions calculated using Eq. (28) for the same parameters $\sigma$, $\lambda$ and $\varepsilon$. Right: Monte Carlo simulation of time distributions for $^{3}$H and $^{14}$C and the time distributions calculated using Eq. (29).

In order to validate the equations describing the cross-correlation distribution as well as to better visualize its shape for different initial parameters we developed a dedicated Monte Carlo code. The code generates realistic list-mode files from a two or three PMT detector system measuring a LS-source. In a first step the code reads the input parameters from the user, which are:

- the parameters of the liquid scintillator used to calculate the ionization quenching function (Z/A, density and mean excitation energy);
- the energy spectrum of the radionuclide and its activity
- the parameters describing the response of the PMTs ($\sigma , \mu , \varepsilon$);
- the decay constant of the scintillator $\lambda$ and the figure of merit $\varphi$;
- the number of decays to be simulated.

After the initial step, the code enters in a loop where, on each iteration, an energy is sampled from the energy spectrum of the radionuclide. The effective deposited energy is calculated, after taking into account Birks’ ionization quenching function. The number of detected photons is calculated as the product of the effective energy and the figure of merit. The timestamp of each detected photon is sampled from an exponential distribution with the decay constant of the scintillator. For each photon, the PMT that detects it is selected from a weighted uniform distribution, where the weights are the relative PMT efficiencies. The photons in each PMT are sorted according to their timestamp and the primary events are written in a list-mode file. Then the loop cycles from the beginning. The list-mode files can then be analyzed with the same software that is used for the analysis of list mode data from real measurements.

The validation of Eqs. (28) and (29) is shown in Fig. 11. The figure on the left shows different cross-correlation distributions for a predefined average number of photons from 2.5 to 20.5, illustrating the change in the shape of the distribution with increasing number of photons. In particular it can be seen how the distribution becomes narrower and its value at zero-time difference increases. The comparison between the Monte Carlo simulation and the analytical equation taking into account the spectrum of the radionuclide can be seen on the right. The Monte Carlo data was generated for $^3$H and $^{14}$C for two different figures of merit. An excellent agreement can be seen between the simulated data and Eq. (29).

### Curve fitting procedure

The experimentally obtained cross-correlation distributions are fitted with Eq. (29) and its parameters are estimated. The fitting was performed with a custom Python program and the minimization of the $\chi ^2$ was done using the Nelder–Mead algorithm provided by the scipy package^23^. The calculation of Eq. (29) is performed by a different pre-compiled code that accepts $\varphi , \lambda , \varepsilon , \mu , \sigma$ and the parameters of the ionization quenching formula and returns the cross-correlation distribution for a specified time range and with a given time step. This is done to shorten the computation time and to allow the parallelization of the calculation. The Poisson distribution summation is limited to Poisson coefficients larger than $10^{-4}$. A typical fitting of the analytical equation to experimental data takes approximately 30 s on a portable computer with a processor with 4 physical cores at 3.4 GHz.
